# PSC-derived Galectin-1 inducing epithelial-mesenchymal transition of pancreatic ductal adenocarcinoma cells by activating the NF-κB pathway

**DOI:** 10.18632/oncotarget.21212

**Published:** 2017-09-23

**Authors:** Dong Tang, Jingqiu Zhang, Zhongxu Yuan, Hongpeng Zhang, Yang Chong, Yuqin Huang, Jie Wang, Qingquan Xiong, Sen Wang, Qi Wu, Ying Tian, Yongdie Lu, Xiao Ge, Wenjing Shen, Daorong Wang

**Affiliations:** ^1^ Department of General Surgery, Institute of General Surgery, Northern Jiangsu Province Hospital, Clinical Medical College, Yangzhou University, Yangzhou, P.R. China; ^2^ Department of General Surgery, Anhui No. 2 Provincial People’s Hospital, Hefei, Anhui Province, P.R. China; ^3^ Department of General Surgery, The First Affiliated Hospital of Nanjing Medical University, Nanjing, P.R. China; ^4^ Department of Clinical Medicine, Medical College of Yangzhou University, Yangzhou, P.R. China

**Keywords:** Galectin-1, invasion, metastasis, pancreatic ductal adenocarcinoma (PDAC), epithelial-mesenchymal transition (EMT)

## Abstract

Galectin-1 has previously been shown to be strongly expressed in activated pancreatic stellate cells (PSCs) and promote the development and metastasis of pancreatic ductal adenocarcinoma (PDAC). However, the molecular mechanisms by which Galectin-1 promotes the malignant behavior of pancreatic cancer cells remain unclear. In this study, we examined the effects of Galectin-1 knockdown or overexpression in PSCs co-cultured with pancreatic cancer (PANC-1) cells. Immunohistochemical analysis showed expression of epithelial-mesenchymal transition (EMT) markers and MMP9 were positively associated with the expression of Galectin-1 in 66 human PDAC tissues. In addition, our *in vitro* studies showed PSC-derived Galectin-1 promoted the proliferation, invasion, and survival (anti-apoptotic effects) of PANC-1 cells. We also showed PSC-derived Galectin-1 induced EMT of PANC-1 cells and activated the NF-кB pathway *in vitro*. Our mixed (PSCs and PANC-1 cells) mouse orthotopic xenograft model indicated that overexpression of Galectin-1 in PSCs significantly promoted the proliferation, growth, invasion, and liver metastasis of the transplanted tumor. Moreover, Galectin-1 overexpression in PSCs was strongly associated with increased expression of EMT markers in both the orthotopic xenograft tumor in the pancreas and in metastatic lesions of naked mice. We conclude that PSC-derived Galectin-1 promotes the malignant behavior of PDAC by inducing EMT via activation of the NF-κB pathway. Our results suggest that targeting Galectin-1 in PSCs could represent a promising therapeutic strategy for PDAC progression and metastasis.

## INTRODUCTION

Pancreatic ductal adenocarcinoma (PDAC) remains one of the most lethal diseases worldwide; it is the fourth leading cause of cancer death, with a median survival time of approximately 6 months and a 5-year survival rate of less than 5% [1]. At diagnosis, 50%–60% of patients have advanced disease with distant metastases and of the ∼10% of patients who undergo a curative resection, many will relapse with distant and/or locoregional metastases [2, 3].

The poor prognosis of PDAC is due to its aggressive growth and rapid development of distant metastases, as well as the low rate of eligibility for surgical resection and chemoradiation resistance [4]. Histopathologically, PDAC is often accompanied by a dense desmoplastic reaction [5], which constitutes the characteristic stromal structure of the cancer. This desmoplastic microenvironment of PDAC, forming approximately 80% of the tumor mass, is an active player in carcinogenesis [6]. One of the driving cellular components of the desmoplastic response in PDAC is activated pancreatic stellate cells (PSCs) [7, 8].

Increasing evidence indicate that the interaction between activated PSCs and pancreatic cancer cells plays an important role in the development and progression of PDAC. By producing high levels of cytokines, chemotactic factors, growth factors, and excessive extracellular matrix (ECM) proteins, PSCs create desmoplasia and a hypoxic microenvironment that promotes the initiation, development, evasion of immune surveillance, invasion, metastasis, and resistance to chemoradiation of pancreatic cancer cells [9–14]. Indeed, targeting the interaction between PSCs and pancreatic cancer cells in the tumor microenvironment may represent a novel therapeutic approach to advanced PDAC [4, 6, 15]. However, despite increasing interest, there is lack of critical understanding of the specific roles and effects of PSCs in PDAC.

Galectin-1 (a member of the galectin family of β-galactoside-binding proteins) is a 28-kDa homodimer, with each subunit possessing two β-galactoside-binding sites [14]. Galectin-1 has a variety of functions, including cell growth, and cell-cell and cell-matrix interactions [16]. Increasing evidence indicates that *Galectin-1* expression is dysregulated in cancer, and may contribute to tumor invasion and metastasis [17], angiogenesis [18], and protecting tumors from host immune responses [14, 19]. In addition, a previous study demonstrated that Galectin-1 could function in the desmoplastic reaction that occurs around PDAC cells [20]. Indeed, recently, it has been shown that *Galectin-1* is strongly expressed in isolated culture-activated PSCs and induces chemokine production and proliferation of PSCs [20, 21]. Another study showed that Galectin-1 was mainly expressed in activated PSCs in PDAC, and significantly promotes growth, proliferation, and progression of PDAC [22, 23].

Galectin-1 in PSCs has been shown to activate ERK, JNK, AP-1, and NF-кB to promote cell proliferation and migration [21, 24]. The MAP kinase (MAPK) pathway (of which ERK and JNK are members) and the NF-кB pathway contributed to the epithelial-mesenchymal transition (EMT) of cancer cells. Furthermore, recent studies indicated that Galectin-1 triggered EMT in human gastric cancer and hepatocellular carcinoma cells [25, 26], thereby promoting tumor invasion and metastasis. Although Galectin-1 has been shown to be strongly expressed in PDAC tissues [14, 23], precisely how PSC-derived Galectin-1 triggers EMT has not yet been elucidated.

This study aimed to investigate the effects of *Galectin-1* expression in primary PSCs on the behavior of PDAC cells both *in vitro* and *in vivo*. In particular, we aimed to determine whether PSC-derived Galectin-1 promotes the proliferation, invasion, and metastasis of PDAC cells by inducing EMT through activating the NF-кB pathway, and to clarify the function of PSC-derived Galectin-1 as abridge between the desmoplastic stroma and cancer cells in the tumor microenvironment of PDAC. And this research could provide us potential therapeutic strategy targeting PSC-derived Galectin-1 for PDAC.

## RESULTS

### PSC-derived Galectin-1 expression is positively correlated with the expression of EMT markers and MMP9 in human PDAC tissues

Our previous studies have shown Galectin-1 expression in PSCs increases with the degree of malignancy of pancreatic cancer [14, 23]. Here we evaluated the correlation between PSC-derived Galectin-1 expression and changes in the expression of EMT markers and the invasive ability of PDAC cells.

**Figure 1:**
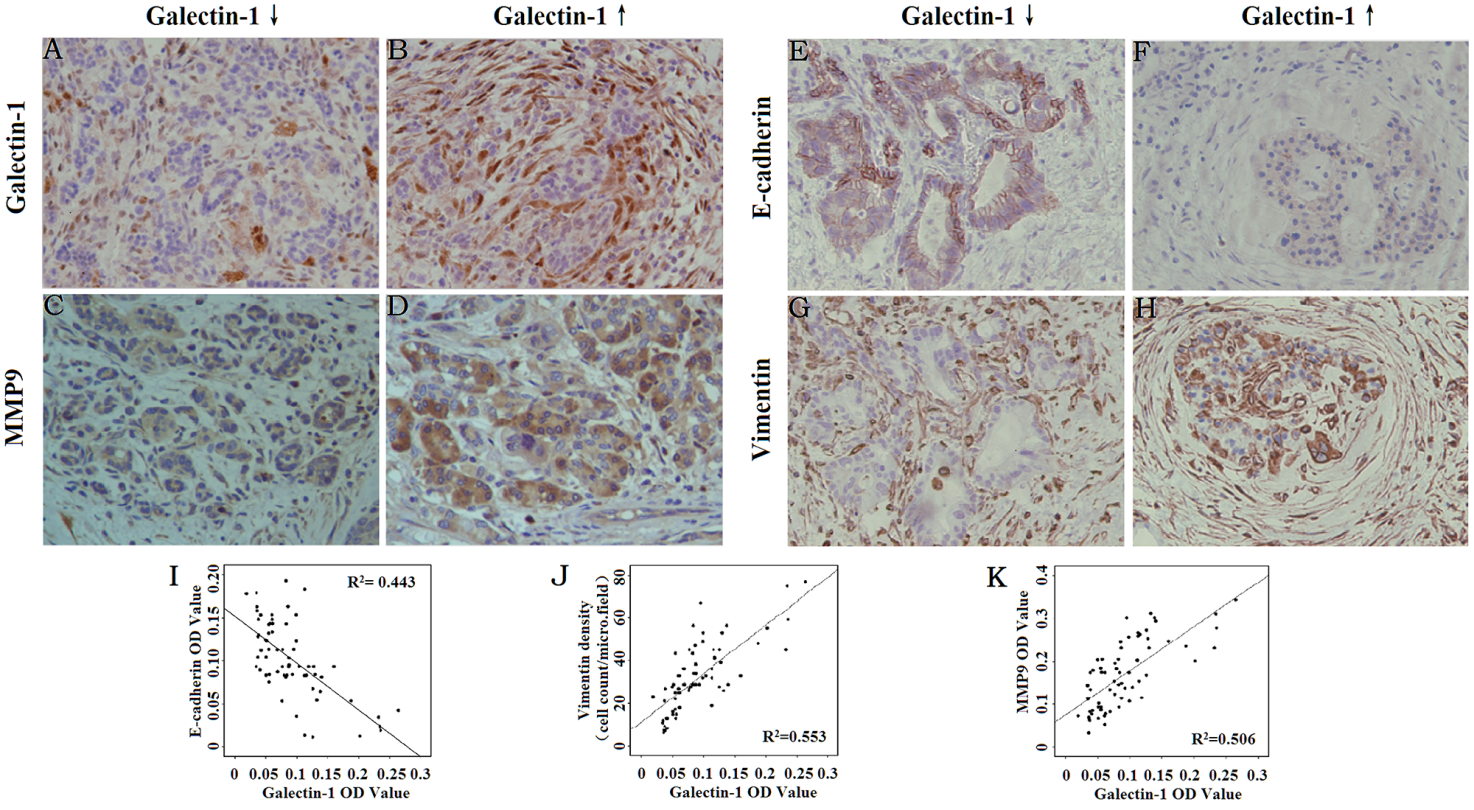
Correlation between Galectin-1 expression and the expression of EMT markers and MMP9 Immunohistochemical staining for Galectin-1 **(A, B)**, MMP9 **(C, D)**, E-cadherin **(E, F)**, and Vimentin **(G, H)** in PDAC tissues. Original magnification: ×200. **(I)** Correlation analysis of the mean density of Galectin-1 and E-cadherin. **(J)** Correlation analysis of the mean density of Galectin-1 and Vimentin. **(K)** Correlation analysis of the mean density of Galectin-1 and MMP9.

We found Vimentin (a mesenchymal cell marker) and MMP9 expression were positively correlated with Galectin-1 expression, while E-cadherin (an epithelial cell marker) expression was negatively correlated with the Galectin-1 expression in PDAC (Figure 1). In particular, with enhanced PSC-derived Galectin-1 expression, Vimentin and MMP9 expression increased in the cytoplasm of the stromal area around the PDAC cells and E-cadherin expression decreased on the cancer cell membrane (Figure 1A–1H). These results suggest that PSC-derived Galectin-1 promotes the invasion of PDAC by inducing the EMT of cancer cells.

### Galectin-1 overexpression and knockdown in PSCs and PSCs co-cultured with PANC-1

We performed lentiviral hGalectin-1 and shRNA-Galectin-1 transduction (as described previously [14]) to obtain PSCs with: (1) normal Galectin-1 expression (wt), (2) an overexpression control (GFP), (3) Galectin-1 overexpression (over), (4) a control shRNA-scrambled (scr), (5) Galectin-1 knockdown shRNA #1 (sh-1), and (6) Galectin-1 knockdown shRNA #2 (sh-2). Galectin-1 expression in PSCs was confirmed by qRT-PCR and Western blotting (Supplementary Figure 1D–F).

To evaluate the direct effect of PSCs on PANC-1 cells, we co-cultured PSCs and PSNC-1 cells together on a monolayer. Immunohistochemical staining showed the over-Galectin-1 PSCs group had significant staining (Supplementary Figure 1A), the wt-Galectin-1 PSCs group had moderate staining (Supplementary Figure 1B), and the sh-Galectin-1 PSCs group had negative staining (Supplementary Figure 1C), indicating Galectin-1 was upregulated and downregulated successfully.

### PSC-derived Galectin-1 promotes the proliferation, invasion, and survival of PANC-1 *in vitro*

The high expression of Galectin-1 in PDAC, along with its positive relation with the expression of EMT markers and MMP9 expression, prompted us to investigate whether PSC-derived Galectin-1 functions as a tumor promoter. We evaluated the effect of PSC-derived Galectin-1 on the proliferation of the pancreatic cancer cell line, PANC-1.

**Figure 2:**
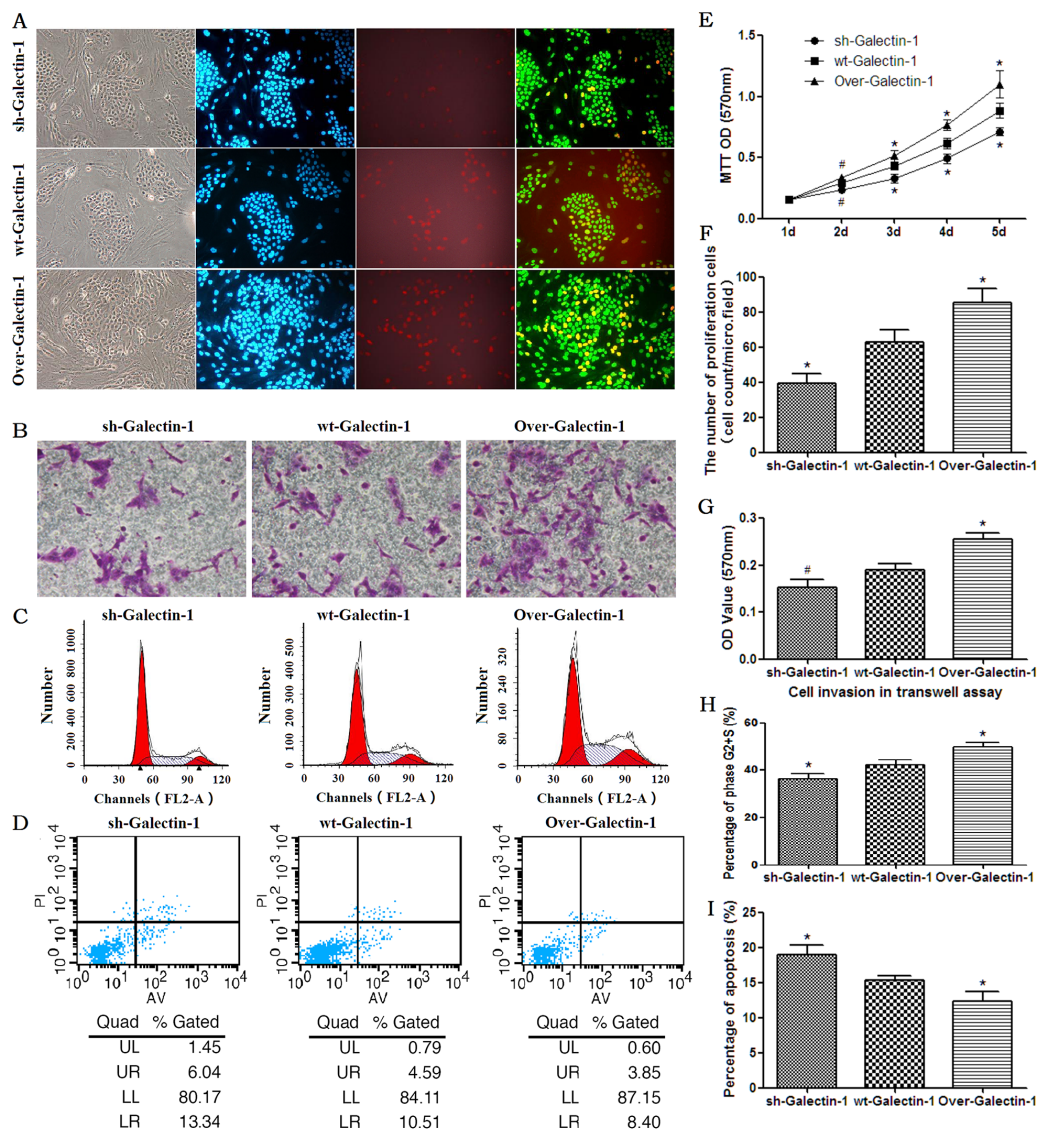
Effect of PSC-derived Galectin-1 on the proliferation and migration ability of PANC-1 **(A)** Proliferating capability of PANC-1 promoted by PSCs as evaluated by EdU incorporation (A). The number of proliferating cells (EdU cell count per micro field) is shown in **(F)** and cell growth over 5 days (measured using MTT assays) is shown in **(E)**. **(B)** Invasion ability of PANC-1 promoted by PSCs as detected by the transwell invasion assay. The OD value of each group of invaded PANC-1 cells is shown in **(G)**. **(C)** PSCs derived Galectin-1 promotes the proliferative activity (G2+S-phase fraction) of PANC-1 cells. Bar-graph representation of the G2+S-phase fraction cells in each group is shown in **(H)**. **(D, I)** PSCs derived Galectin-1 has an anti-apoptotic effect on PANC-1 *in vitro*. All experiments were repeated three times. *p < 0.05, **p < 0.01, #p > 0.05 vs. wt-Galectin-1 PSCs.

Using an EdU incorporation cell proliferation assay, we found an increased proportion of proliferating PANC-1 cells co-cultured with PSCs overexpressing Galectin-1 in the same monolayer compared with wild type PSCs (Figure 2A–2F). The opposite result was observed in PSCs in which Galectin-1 had been knocked down. The MTT assay also confirmed proliferation of PANC-1 cells was promoted by PSCs overexpressing Galectin-1 in the transwell culture system (Figure 2E).

We further examined whether endogenous Galectin-1 derived from PSCs affects the invasion ability of PANC-1. With the presence of PSCs in the lower transwell chambers, the invasion ability of PANC-1 was significantly enhanced in PSCs overexpressing Galactin-1 (p = 0.003 vs. control), and was significantly decreased in those with wild type Galectin-1 expression (p = 0.04 vs. control) (Figure 2B, 2G). This indicates that high expression of Galectin-1 in PSCs contributes to the invasive ability of PDAC cells.

To determine whether the PSC-derived Galectin-1 induces the proliferative activity of PANC-1 cells in the co-culture system, we analyzed the G2+S phase fraction of PANC-1 by flow cytometry, and quantified the percentages of cells in the apoptotic, G1, S, and G2 phases. The mean percentage of PANC-1 cells in the G2+S phase in the group containing PSCs overexpressing Galectin-1 was significantly higher than those with wild type expression (49.85 ± 1.68% vs. 42.13 ± 2.17%, respectively; p = 0.008). However, mean percentage of PANC-1 cells in the G2+S phase in the group containing PSCs with Galectin-1 knocked down (36.23 ± 2.01%) was significantly less those with wild type expression (p = 0.026) or with Galactin-1 overexpression (p = 0.001) (Figure 2C, 2H).

We next assessed the effect of PSC-derived Galectin-1 on the anti-apoptotic ability of PANC-1 in the co-culture system. We found overexpression of Galectin-1 in PSCs significantly decreased apoptosis of PANC-1 cells compared to those with wild type expression (p = 0.029); alternatively, knockdown of Galectin-1 expression in PSCs increased apoptosis of PANC-1 cells compared to wt-Galectin-1 PSCs (Figure 2D, 2I; p = 0.014). This suggests that PSC-derived Galectin-1 has an anti-apoptotic effect on PANC-1.

### PSC-derived Galectin-1 induces changes in cell phenotype and expression of EMT markers in PANC-1 partly through the NF-кB pathway

**Figure 3:**
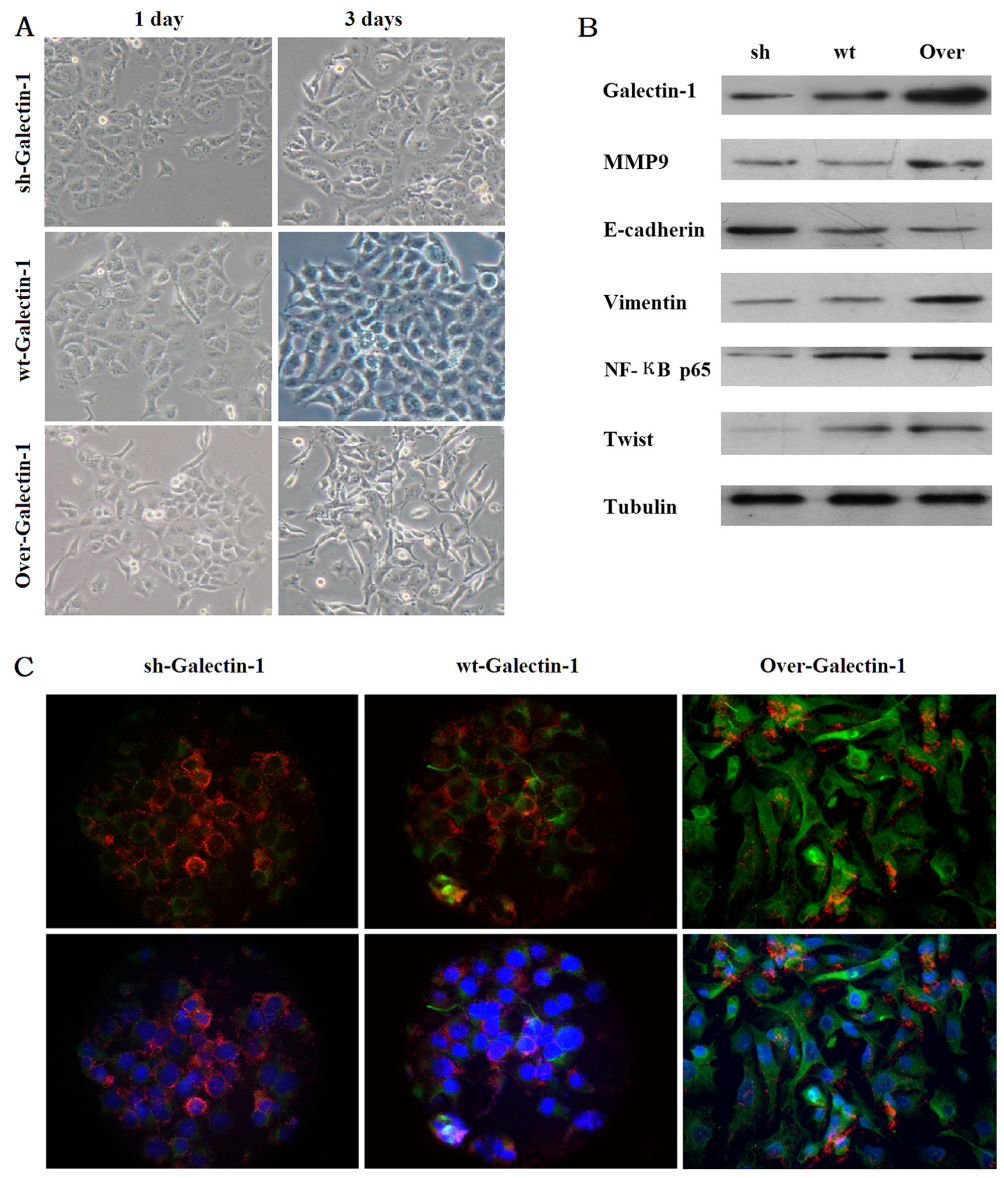
Effect of PSCs derived Galectin-1 expression on PANC-1 phenotype and expression changes **(A)** PANC-1 cell phenotype changes resulting from different levels of Galectin-1 expression. **(B)** Western blot analysis of MMP9, EMT markers, NF-кB, and core transcriptional factor Twist expression, according to Galectin-1 expression in PSCs. **(C)** Immunofluorescence confirming that the EMT markers change with Galectin-1 expression in PSCs. The red represents E-cadherin expression, the green represents Vimentin expression, and the blue represents Hoechst 33342 (DNA/nuclei stain). Original magnification: 200×.

PANC-1 cells normally exhibit an epithelioid-like phenotype. We examined the phenotype of PANC-1 cells at 1 and 3 days after co-culture with PSCs. After co-cultured with PSCs overexpressing Galectin-1 for 3 days, the PANC-1 cells acquired a spindle-shaped morphology and grew separately (Figure 3A). However, when co-cultured with PSCs expressing wild type or knocked down Galectin-1 for 3 days, the PANC-1 cells still exhibited epithelioid-like phenotype and proliferated in clusters (Figure 3A).

With respect to EMT markers, overexpression of Galectin-1 in PSCs increased Vimentin expression but decreased E-cadherin levels, while Galectin-1 knockdown in PSCs decreased Vimentin expression and increased E-cadherin levels in PANC-1 cells (Figure 3B). In addition, PSCs overexpressing Galectin-1 promoted the invasion ability of PANC-1 by increased the MMP9 expression; the opposite result was observed in the group containing PSCs with Galectin-1 knockdown (Figure 3B).

We also found that expression of NF-κB p65 and the core transcriptional factor Twist in PANC-1 cells were enhanced in the group containing PSCs overexpressing Galectin-1, and decreased in the Galectin-1 knockdown group (Figure 3B). Our immunofluorescence results agreed with the western blot analysis: Galectin-1 overexpressing PSCs decreased E-cadherin fluorescence and upregulated Vimentin fluorescence in PANC-1 cells, while Galectin-1 knockdown PSCs increased E-cadherin fluorescence and downregulated Vimentin fluorescence in PANC-1 cells compared to Galectin-1 wild type PSCs (Figure 3C). These results confirm that PSC-derived Galectin-1 induces the EMT of PANC-1, in part by activating the NF-кB pathway.

### PSC-derived Galectin-1 promotes the malignant behavior of PDAC by inducing the EMT of cancer cells *in vivo*

**Figure 4:**
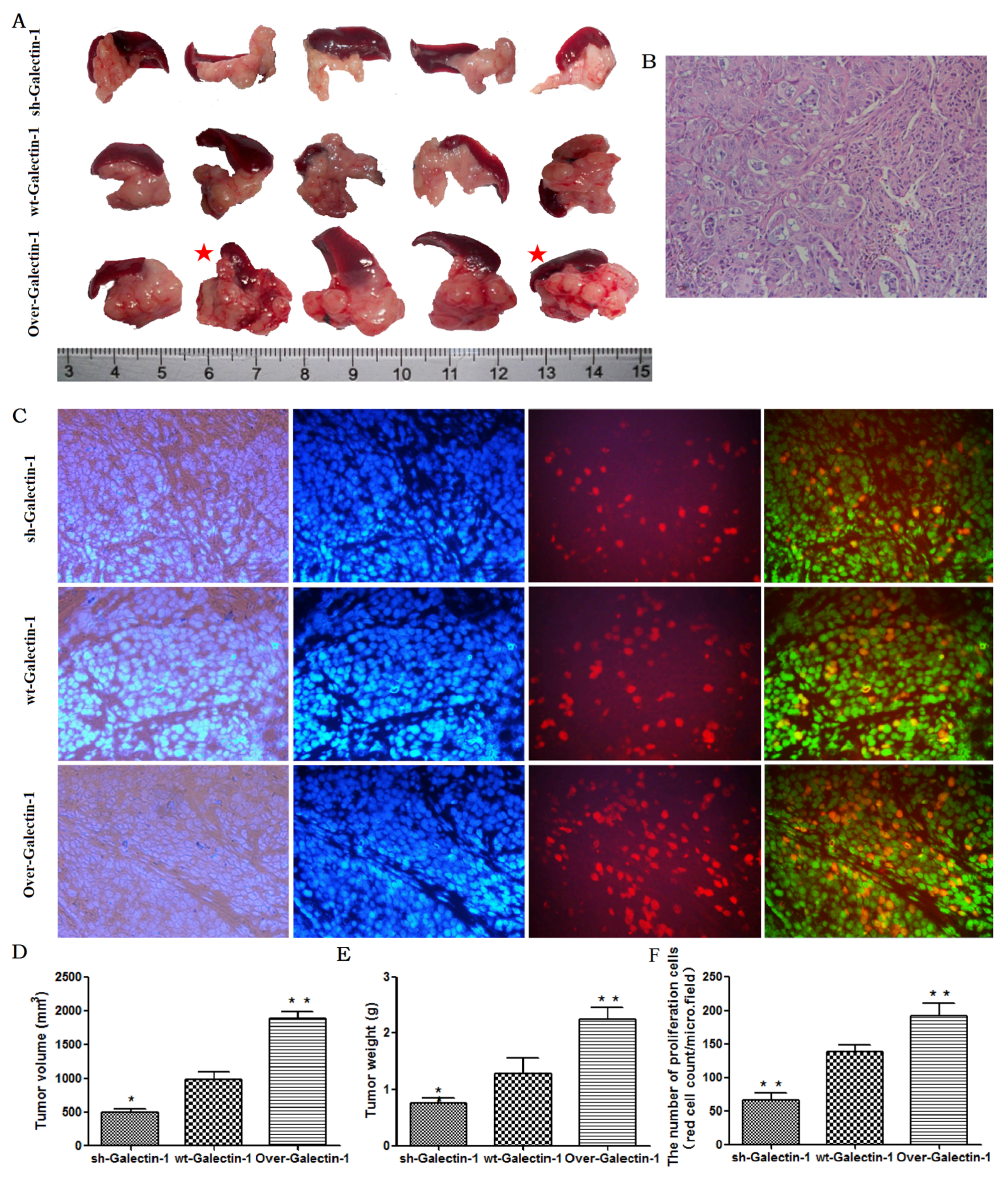
Effect of PSC-derived Galectin-1 on *in vivo* orthotopic xenograft establishment and growth **(A)** PANC-1 mixed with PSCs were implanted orthotopically into the pancreas of nude mice (n = 5). The mice were sacrificed and the xenografts were removed on day 30 after cell implantation. The red star represent cases with liver metastasis. **(B)** H&E staining of samples of orthotopic xenografts in the pancreas of nude mice. **(C)** Proliferating capability of the orthotopic xenograft was evaluated using the EdU incorporation assay, and the number of EdU positive cells per micro field is shown in **(F)**. Tumor volume **(D)** and weight **(E)** is expressed as the mean ± SE. *p < 0.05, **p < 0.01, #p > 0.05 vs. wt-Galectin-1 PSCs.

To validate the effect of PSC-derived Galectin-1 on PDAC cells *in vivo*, we used a mouse orthotopic xenograft model, in which PANC-1 cells mixed with PSCs were implanted orthotopically into the pancreas of mice, and the pathological results were evaluated by Hematoxylin and Eosin (H&E) staining (Figure 4B). We found Galectin-1 overexpressing PSCs significantly promoted tumor growth, while Galectin-1 knockdown PSCs reduced the tumor size compared to the control mice (p < 0.05). Tumors from mice containing Galectin-1 knockdown PSCs reached a volume of 501.60 ± 38.50 mm^3^ and a weight of 0.76 ± 0.09 g at 30 days after implantation; tumors in the control group (wt-Galectin-1 PSCs) reached a volume of 983.20 ± 111.59 mm^3^ and weight of 1.27 ± 0.27 g; and tumors from mice with Galectin-1 overexpressing PSCs only reached a volume of 1881.00 ± 102.19 mm^3^ and weight of 2.24 ± 0.22 g (Figure 4A, 4D, 4E).

When the sections from all the tumors were analyzed by the EdU incorporation cell proliferation assay, a higher number of proliferating cells were found in tissues from mice with Galectin-1 overexpressing PSCs compared with the control group (wt-Galectin-1 PSCs), while the opposite result was observed in tissues from mice containing Galectin-1 knockdown PSCs (Figure 4C). These results are still consistent with the earlier findings *in vitro*.

In addition, 40% (2/5) of the group of mice containing Galectin-1 overexpressing PSCs exhibited liver metastasis, while no metastases were observed in mice containing Galectin-1 wild type or knockdown PSCs. Together, these results indicate PSC-derived Galectin-1 promotes tumor establishment, growth, and metastasis.

**Figure 5:**
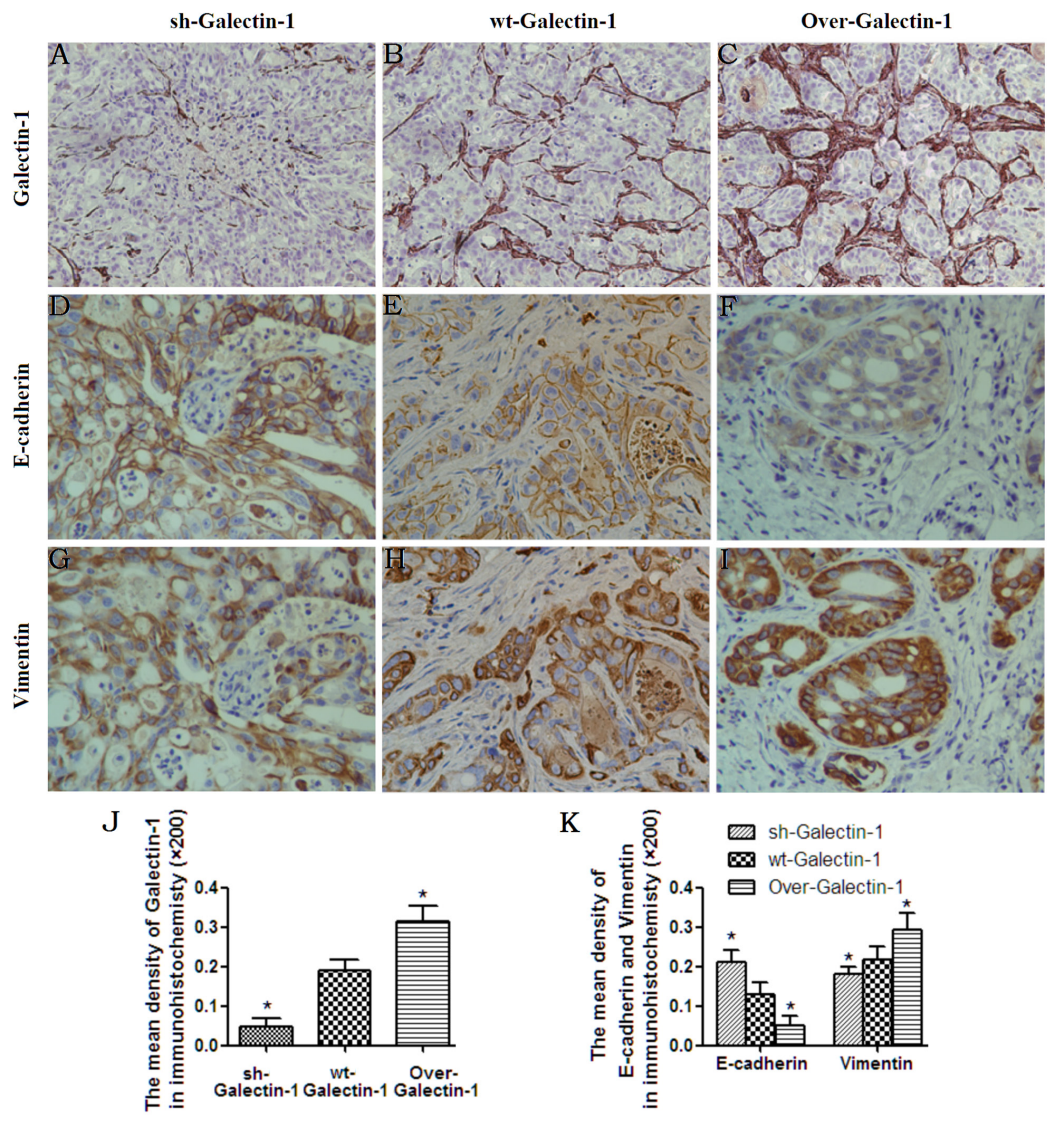
Effect of PSC-derived Galectin-1 on EMT of cancer cells *in vivo* Immunohistochemistry staining for Galectin-1 **(A–C)**, E-cadherin **(D–F)**, and Vimentin **(G–I)** expression in orthotopic xenograft tissues. Original magnification: ×200. **(J)** The mean density of Galectin-1 in the different orthotopic xenograft tissues. **(K)** The mean density of E-cadherin and Vimentin in the different orthotopic xenograft tissues. Original magnification: 200×. *p < 0.05, **p < 0.01, #p > 0.05 vs. wt-Galectin-1 PSCs.

We next asked whether overexpression of Galectin-1 in PSCs promotes the invasion of PDAC cells by inducing EMT *in vivo*. All orthotopic tumors from the mice pancreas were analyzed by immunohistochemical staining. We found that tumors from mice containing Galectin-1 overexpressing PSCs had higher levels of Galectin-1 in the PSCs around the PANC-1 cells than controls (wt-Galectin-1 PSCs) (Figure 5B, 5C, 5J). Moreover, very weak Galectin-1 staining was observed in the tumors from mice containing Galectin-1 knockdown PSCs (Figure 5A, 5J). We also found Galectin-1 overexpressing PSCs increased Vimentin expression and decreased E-cadherin levels (Figure 5F, 5I, 5K), while Galectin-1 knockdown PSCs decreased Vimentin expression and increased E-cadherin levels in PANC-1 cells (Figure 5B, 5K). Consistent with our *in vitro* findings, the control mice (with wt-Galectin-1 PSCs) had moderate Vimentin and E-cadherin staining. These results indicate PSC-derived Galectin-1 promotes the invasion of PANC-1 through EMT.

**Figure 6:**
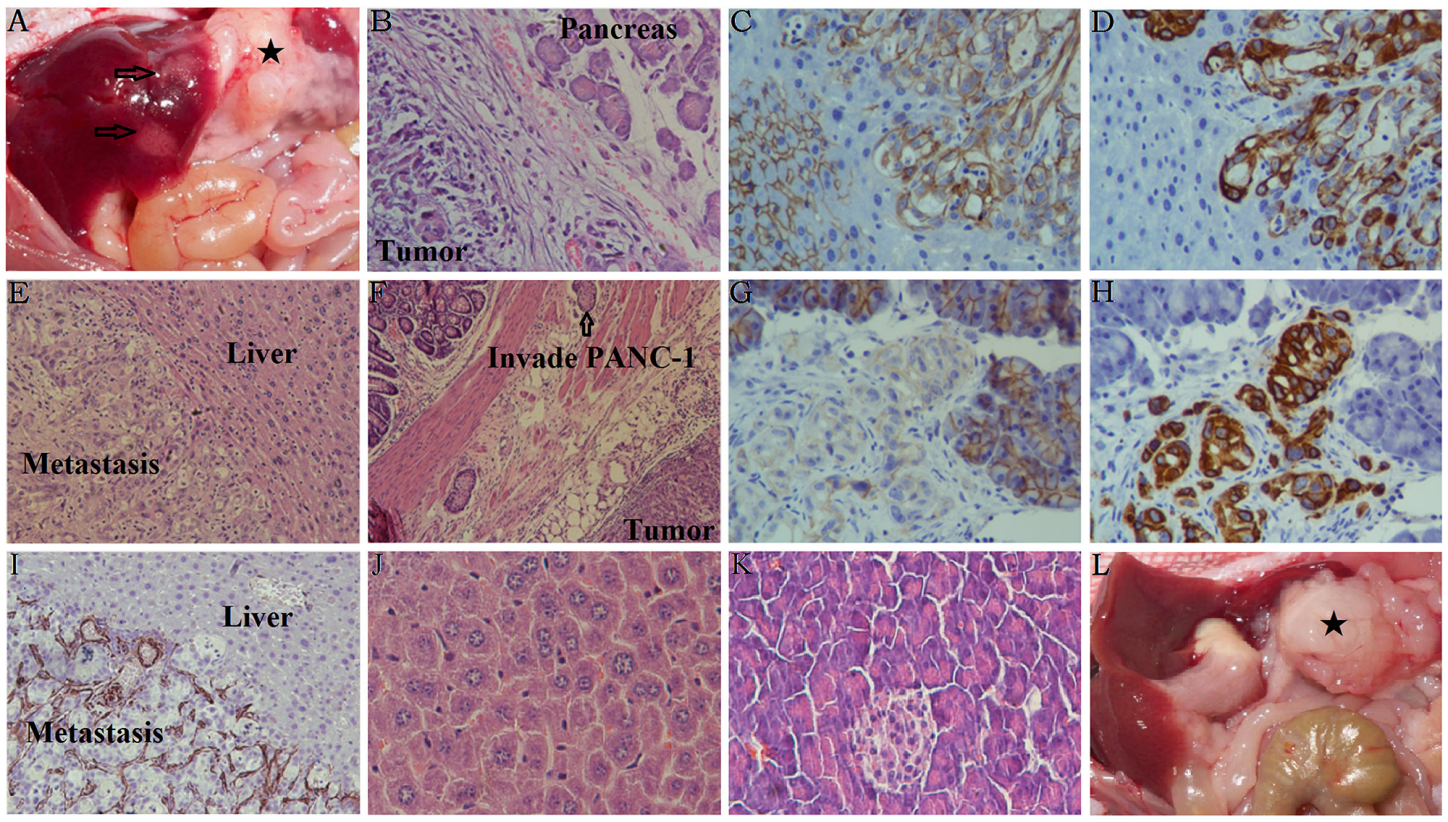
Characteristics of orthotopic tumors in mice with or without liver metastasis **(A)** The black star represents the orthotopic xenograft tumor containing Galectin-1 overexpressing PSCs mixed with PANC-1, and the arrows represent the liver metastasis. **(B)** H&E staining of the orthotopic xenografts in the mice pancreas. **(C)** Decreased E-cadherin staining was observed in the liver metastasis. **(D)** Increased Vimentin staining was observed in the liver metastasis. **(E)** H&E staining of the liver metastases. **(F)** H&E staining of the stomach in mice with Galectin-1 overexpressing PSCs mixed with PANC-1. **(G)** Decreased E-cadherin staining was observed in the orthotopic xenografts in the mice pancreas. **(H)** Increased Vimentin staining was observed in the orthotopic xenografts in the mice pancreas. **(I)** Galectin-1 staining was observed in the liver metastasis. **(J)** H&E staining of the normal mice liver as a control. **(K)** H&E staining of the normal mice pancreas as a control. **(L)** The black star represents the orthotopic xenograft tumor containing wt-Galectin-1/sh-Galectin-1 PSCs mixed with PANC-1; in these cases, no liver metastasis was observed.

Invading PANC-1 cells were also found in the pancreas (Figure 6B), the wall of the stomach (Figure 6F), and in the liver (Figure 6A, 6E). Immunohistochemical staining showed that increased Vimentin expression and decreased E-cadherin levels were found in tumors marginal of the pancreas (Figure 6G, 6H) and in the liver metastasis (Figure 6C, 6D) from mice with Galectin-1 overexpressing PSCs. Strong staining of Galectin-1 was found in the focus of liver metastases (Figure 6I); however, whether Galectin-1 expressing stromal cells are recruited by the metastastic cancer cells or the metastatic PSCs move together with the cancer cells requires further investigation. In short, we confirm that PSC-derived Galectin-1 induces the EMT of PANC-1 cells *in vivo* and significantly promotes the malignant behavior of invasion and metastasis.

## DISCUSSION

The interaction between pancreatic cancer cells and PSCs is receiving increasing attention. This study evaluated the mechanism of how PSC-derived Galectin-1 promotes malignancy in PDAC. We found Galectin-1 protein expression was positively correlated with the expression of EMT markers and MMP9 in human PDAC tissues. PSC-derived Galectin-1 promoted the proliferation, invasion, and survival of a pancreatic cancer cell line (PANC-1). In addition, PSC-derived Galectin-1 induced EMT of PANC-1 cells, in part through activation of the NF-кB pathway*in vitro*. Furthermore, we found PSC-derived Galectin-1 significantly promoted invasion and metastasis (by inducing EMT) of both orthotopic and metastatic tumors *in vivo*. Together, our results suggest that PSC-derived Galectin-1 might serve as a novel biomarker to predict the risk for invasion and metastasis in PDAC.

An intense stromal desmoplastic reaction surrounding the cancer cells is a consistent hallmark of PDAC and has emerged as a major contributing factor for chemotherapy resistance [27]. In particular, Galectin-1 (which is highly expressed in PSCs in PDAC) plays a key role in this desmoplastic reaction, with elevated Galectin-1 expression highly correlated to tumor histology, stage, and poor prognosis [20, 21, 23, 28]. Indeed, lower Galectin-1 expression is associated with longer survival in PDAC [29].

Previous studies have shown that overexpression of Galectin-1 in PSCs induced T cell apoptosis, anergy, and Th2 cytokine secretion [14, 30], and promoted the proliferation, invasion, and metastasis of pancreatic cancer cells [23]. Together these findings indicate Galectin-1 is involved in PSCs cross-talk in PDAC, and provide a preclinical rationale for targeting Galectin-1 as a tumor microenvironment-based therapeutic strategy [27]. However, few studies have elucidated the precise role of Galectin-1 in the pancreatic cancer-stroma interaction. Other studies have shown PSCs could promote EMT in pancreatic cancer cells, suggesting a novel mechanism by which PSCs contribute to the aggressive behavior of pancreatic cancer cells [31–33]. According to several recent studies, Galectin-1 could activate FAK/P13K/ATK signaling, hedgehog signaling, and bind β1 integrin, inducing the EMT process in cancer cells [34–36]. Indeed, as Galectin-1 was recently reported to trigger EMT and tumor progression in human hepatocellular carcinoma [25], gastric cancer [26], and colon cancer [37], we hypothesized that it may also induce EMT in pancreatic cancer, which is the foundation of this research.

In this study, we showed enhanced Galectin-1 expression was positively correlated with Vimentin and MMP9 expression and negatively correlated with E-cadherin expression, indicating that enhanced Galectin-1 expression may promote the EMT and the invasion ability of PDAC cells. We also found that when PSCs were co-cultured with PANC-1, PSC-derived overexpression of Galectin-1 accelerated the proliferation, invasion, and survival of PANC-1 cells, and promoted EMT of PANC-1 cells *in vitro*. On the contrary, knockdown of Galectin-1 in PSCs dramatically reduced cell proliferation and invasion ability, increased E-cadherin expression, and decreased Vimentin expression. These results were completely verified *in vivo* in the orthotopic xenograft models. In particular, PSC-derived overexpression of Galectin-1 promoted the invasion and metastasis of pancreatic cancer cells into the wall of the stomach and liver by inducing EMT. Taken together, PSC-derived Galectin-1 elevates the malignant behavior of PDAC cells by promoting growth, invasiveness, and metastasis, in part by inducing EMT.

When PSCs and PANC-1 were co-cultured together, PSC-derived overexpression of Galectin-1 significantly promoted the expression of NF-κB p65, and the core transcriptional factor Twist; while reduction of Galectin-1 expression in PSCs downregulated NF-κB p65 and Twist expression. NF-κB is a transcription factor that has previously been shown to play a key role in the induction of EMT in pancreatic cancer cells, thereby facilitating the invasiveness and metastasis of PDAC [38]. Indeed, the expression of NF-κB is associated with reduced E-cadherin expression (an epithelial cell marker) and enhanced expression of Vimentin and N-cadherin (mesenchymal markers), along with increased expression of various transcription factors (e.g. Snail and Twist) [39]. Moreover, another study identified a potential binding site for NF-κB in the basal promoter of Galectin-1, suggesting that NF-κBwas also associated with Galectin-1 regulation [40]. Galectin-1 was also recently shown to promote tumor progression through upregulation of CXCR4 via activation of the NF-κB pathway [41]. In addition, Galectin-1 was shown to induce the production of chemokines by PSCs, mainly via the activation of NF-κB [21]. Together these findings indicate that Galectin-1 promotes cancer progression by activating NF-κB.

Our previous study indicated that the cytokine TGF-β1 could upregulate Galectin-1 expression in PSCs to further promote the proliferation and invasion of pancreatic cancer cells, and support tumor establishment and growth [23]. Together these findings indicate that PDAC cells secrete TGF-β1 in order to stimulate PSCs to overexpress Galectin-1, which in turn stimulates the malignant potency of pancreatic cancer cells, and establishes a vicious cycle of mutually reinforcing mechanisms to sustain the activity of the stromal reaction [4, 42]. These results indicated that PSCs cross talked with pancreatic cancer cells through cytokines and secreted proteins, which played an important role in the development of pancreatic cancer. This current study indicates Galectin-1 further promotes the malignant behavior of PDAC by inducing EMT through activating the NF-κB signaling pathway.

We also showed that PSC-derived overexpression of Galectin-1 promoted liver metastasis in our mouse orthotopic xenograft model, with strong Galectin-1 staining observed in the focus of liver metastases. According to the seed and soil theory of cancer metastasis [43], PANC-1 cells in liver metastases might recruit and activate the local mouse stromal cells (likely hepatic stellate cells in this case) to form the appropriate microenvironment for growth [44, 45]. As Galectin-1 expression has also been reported in hepatic stellate cells, Galectin-1 derived from local hepatic stellate cells may also promote the progression of a metastatic niche [44, 45]. Alternatively, human PSCs may accompany cancer cells to metastatic sites, stimulate angiogenesis, and be able to intravasate/extravasate to and from blood vessels [11]. If this occurred in our orthotopic xenograft model, then primary human PSCs were carried by the PANC-1 cells to the metastatic site, and the primary human PSC-derived Galectin-1 played a sustained role in promoting metastasis and protecting cancer cells from immune cells. Either way, our results indicate Galectin-1 plays a key role of promoter in the invasion and metastatic cascade of PDAC.

In conclusion, our findings show high expression of Galectin-1 in PSCs is related to the progression, invasion, and metastasis of PDAC. PSC-derived Galectin-1 is involved in the cross-talk between the desmoplastic stroma and cancer cells in the tumor microenvironment, and may promote the malignant behavior of PDAC, resulting in poor prognosis [23]. Our results indicate that PSC-derived Galectin-1 induces EMT of cancer cells through activating the NF-κB pathway. Therefore, either Galectin-1 interference or inhibition of the NF-κB pathway may represent promising therapeutic strategies for the treatment of PDAC.

## MATERIALS AND METHODS

### Patients and pancreatic tissues

Information on clinicopathological characteristics of patients, PDAC tissues, histological evaluation of the specimens from the PDAC tissues, and ethics statements have been previously described [14, 23, 30].

### Cells and culture conditions

The isolation, identification, and maintenance of primary human PSCs were described previously [14, 23, 30]. PSCs from passage numbers 2 to 5 were used for all assays. Co-culture experiments were performed as follows: 1) monolayers of naïve PSCs (1 × 10^5^ cells), or PSCs with either Galectin-1 overexpression or knockdown were co-cultured with PANC-1 cells (5 × 10^5^ cells) in the upper and lower chambers of transwell (6-well) plates at 37°C, separately. After co-culture, the *in vitro* invasion assay, flow cytometric analysis, cancer cell phenotype observation, western blotting analysis, and cellular immunofluorescence assays were performed. 2) PSCs (1 × 10^5^cells) with different levels of Galectin-1 expression were co-cultured with PANC-1 cells (2 × 10^5^ cells) in the same layer of the plates. After co-culture, immunohistochemical staining, an EDU proliferation assay, and mixed mouse orthotopic xenograft model experiments were performed.

### Quantitative reverse transcription-polymerase chain reaction

The detailed experimental procedures of total RNA extraction from PSCs with different levels of Galectin-1, quantitative reverse transcription-polymerase chain reaction (qRT-PCR), and the gene-specific primers for human Galectin-1 and β-actin were described previously [14]. All samples were run in triplicate.

### Immunohistochemical staining and evaluation

Immunohistochemical staining, evaluation of the results, and calculation of the mean density of staining was performed as previously described [14, 23]. The primary antibodies were incubated as follows: mouse monoclonal anti-Galectin-1 (sc-166618; 1:200; Santa Cruz Biotechnology, Inc., Santa Cruz, CA, USA), anti-E-cadherin (sc-52327; 1:200; Santa Cruz Biotechnology, Inc., Santa Cruz, CA, USA), anti- Vimentin (sc-6260; 1:200; Santa Cruz Biotechnology, Inc., Santa Cruz, CA, USA), and anti-MMP9 (sc-21733; 1:200; Santa Cruz Biotechnology, Inc., Santa Cruz, CA, USA), anti-NF-кB p65 (sc-8008; 1:200; Santa Cruz Biotechnology, Inc., Santa Cruz, CA, USA), and anti-Twist (sc-81417; 1:200; Santa Cruz Biotechnology, Inc., Santa Cruz, CA, USA).

### Western blotting analysis

Western blotting was performed as previously described [14, 23, 46]. The following antibodies were used: mouse anti-Galectin-1, anti-MMP9, anti-E-cadherin, anti- Vimentin, anti-NF-кB, and anti-Twist antibodies (1:200, Santa Cruz Biotechnology, Inc.).

### Flow cytometry analysis

The effect of PSCs with different expression levels of Galectin-1 on the apoptosis of pancreatic cancer cell line (PANC-1) cells were detected by flow cytometry as previously described [14]. The S-phase fraction (cell cycle analysis) of PANC-1 affected by PSCs with different level of Galectin-1 expression was carried out as described previously [23].

### Preparation and transduction of recombinant lentiviruses

The plasmids used for Galectin-1 overexpression or interference in PSCs were described previously [14]. Briefly, the *hGalectin-1* gene fragment was excised from a human cDNA library and cloned into pHAGE-CMV-MCS-IZsGreen between the BamHI and XhoI restriction enzyme sites, and *Galectin-1* specific oligonucleotides or the scrambled oligonucleotide were ligated into the pLKO.1-puro vector. The viral obtention, infection to PSCs, selection, and identification were described previously [14].

### EdU incorporation assay

Cell proliferation was assessed by the Cell-Light EdU DNA cell proliferation kit (Ribobio, Guangzhou, China), according to the manufacturer’s instructions [47–49].

For testing cell proliferation *in vitro*, PSCs were mixed and co-cultured with PANC-1. After 48 h, the cells were exposed to 50 mM of EdU for additional 4 h at 37°C. The cells were then fixed with 4% formaldehyde for 15 min at room temperature and treated with 0.5% Triton X100 for 20 min at room temperature for permeabilization. After washes with PBS, the cells were treated with 100 mL of 16 ApolloR reaction cocktail for 30 min. Subsequently, the DNA contents of each well of cells were stained with 100 mL of Hoechst 33342 (5 mg/mL) for 30 min and visualized under a fluorescence microscope (Olympus, Japan).

For testing cell proliferation *in vivo*, mice were injected intraperitoneally with 100 μg of EdU in PBS. After 4 h, the formatted tumor tissues (PSCs mixed with PANC-1) in mice pancreas were harvested and the tissue sectioning, immunohistochemistry and observation were performed as previously described [48]. Five groups of confluent cells were randomly selected from each sample image. EdU-positive cells were obtained from the image, and the relative positive ratio was calculated from the average of the five group values.

### *In vitro* proliferation assay

PANC-1 proliferation was determined by methyl thiazolyl tetrazolium (MTT) (sigma, USA) assay as described previously [22]. In total, 2.5 × 10^3^ of PANC-1 cells with over-Galectin-1, sh-Galectin-1, or control were seeded into 96-well plates and cultured with 10% FCS for 12 h until the cells adhered to the plate. The medium was exchanged and absorbance at 570 nm was detected at 24, 48, 72, 96 h in the microtiter plate reader.

### *In vitro* invasion assay

The matrigel invasion assay was used to assess the ability of PANC- 1 cells to penetrate the ECM as previously described [23].

### Immunofluorescence staining

The immunofluorescence staining was performed as described previously [50]. All images were viewed on an Olympus IX71 fluorescent microscope and images were taken under × 10 magnification using the Olympus DP71 camera (Olympus Optical Co. Ltd, Tokyo, Japan).

### Mouse orthotopic xenograft model

The conditions and methods for our animal experiments were carried out as previously described [23, 51]. In brief, 15 mice were randomly divided into three groups (sh-Galectin-1, wt-Galectin-1, and over-Galectin-1). Accordingly, xenografts (n = 5/group) were established by implanting the human pancreatic cell line PANC-1 cells (1 × 10^6^ cells) with PSCs (sh-Galectin-1, wt-Galectin-1, and over-Galectin-1) (5 × 10^5^ cells). After a midline incision of the anterior abdominal wall, the mixed cells (in a total volume of 0.1 mL serum-free medium) were directly injected into the pancreas parenchyma anesthesia by pentobarbital sodium. The mice were euthanized after 30 days and no mortality was observed. The tumor volume was calculated using the formula (width^2^ × length)/2.

### Statistical analysis

Values are expressed as the mean ± standard deviation. All experiments were repeated three times. One-way ANOVA and *t-*tests were used to compare differences between groups. The Pearson correlation test was used to determine the relationship between Galectin-1 and MMP9, E-cadherin, or Vimentin. All *p*-values were two-sided, and *p*-values ≤0.05 were considered to be statistically significant. All statistical analyses were performed using SPSS 13.0 software.

## SUPPLEMENTARY MATERIALS FIGURE



## Abbreviations

PDAC: pancreatic ductal adenocarcinoma
PSCs: pancreatic stellate cells
ECM: excessive extracellular matrix
ERK: extracellular regulated protein kinases
JNK: c-Jun N-terminal kinase
AP-1: activating protein-1
NF-кB: Nuclear factor kappa-B
MAPK: MAP kinase
EMT: epithelial-mesenchymal transition
qRT-PCR: quantitative reverse transcription-polymerase chain reaction
PANC-1: pancreatic cancer cell line
EdU: 5-Ethynyl-2’-deoxyuridine
PBS: phosphate buffer saline
MTT: methyl thiazolyl tetrazolium
USA: United States of America
MMP9: Matrix metallopeptidase 9
shRNA: short hairpin RNA
PI3K: Phosphoinositide 3-kinase
AKT: serine/threoninekinase
CXCR4: C-X-C chemokine receptor type 4
TGF-β1: Transforming growth factor beta 1
