# Correction: PSC-derived Galectin-1 inducing epithelial-mesenchymal transition of pancreatic ductal adenocarcinoma cells by activating the NF-κB pathway

**DOI:** 10.18632/oncotarget.28087

**Published:** 2021-09-28

**Authors:** Dong Tang, Jingqiu Zhang, Zhongxu Yuan, Hongpeng Zhang, Yang Chong, Yuqin Huang, Jie Wang, Qingquan Xiong, Sen Wang, Qi Wu, Ying Tian, Yongdie Lu, Xiao Ge, Wenjing Shen, Daorong Wang

**Affiliations:** ^1^Department of General Surgery, Institute of General Surgery, Northern Jiangsu Province Hospital, Clinical Medical College, Yangzhou University, Yangzhou, P.R. China; ^2^Department of General Surgery, Anhui No. 2 Provincial People’s Hospital, Hefei, Anhui Province, P.R. China; ^3^Department of General Surgery, The First Affiliated Hospital of Nanjing Medical University, Nanjing, P.R. China; ^4^Department of Clinical Medicine, Medical College of Yangzhou University, Yangzhou, P.R. China; ^*^These authors have contributed equally to this work

**This article has been corrected:** Due to accidental placement, some of the images in Figures 2 and 4 are incorrect. In Figure 2B, panels 2 and 3 have been replaced. In Figure 4C, row 3, all four panels have been replaced. The corrected Figures 2 and 4, produced using the original data, are shown below. The authors declare that these corrections do not change the results or conclusions of this paper.

Original article: Oncotarget. 2017; 8:86488–86502. 86488-86502. https://doi.org/10.18632/oncotarget.21212

**Figure 2:**
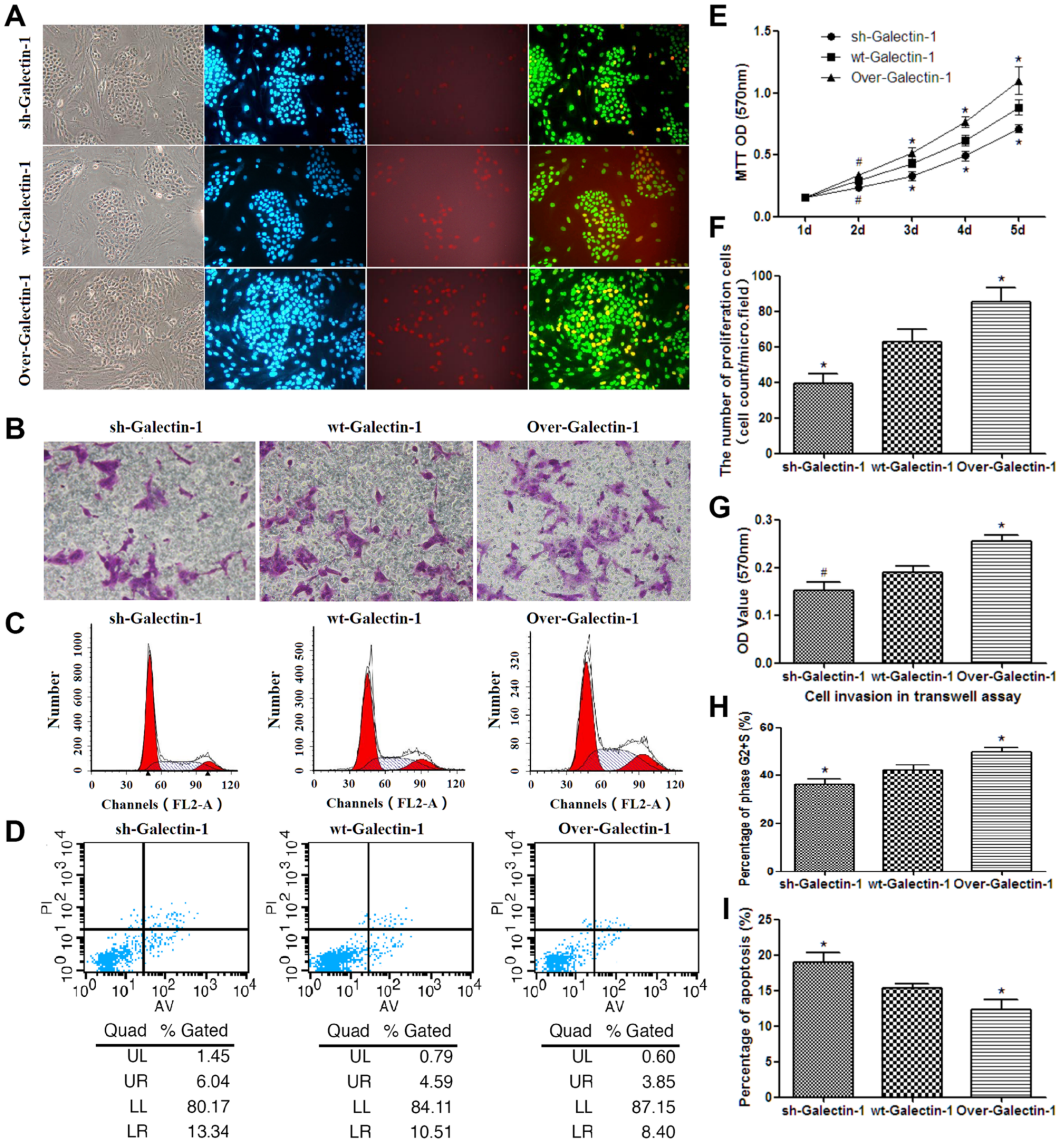
Effect of PSC-derived Galectin-1 on the proliferation and migration ability of PANC-1. (**A**) Proliferating capability of PANC-1 promoted by PSCs as evaluated by EdU incorporation (A). The number of proliferating cells (EdU cell count per micro field) is shown in (**F**) and cell growth over 5 days (measured using MTT assays) is shown in (**E**). (**B**) Invasion ability of PANC-1 promoted by PSCs as detected by the transwell invasion assay. The OD value of each group of invaded PANC-1 cells is shown in (**G**). (C) PSCs derived Galectin-1 promotes the proliferative activity (G2+S-phase fraction) of PANC-1 cells. Bar-graph representation of the G2+S-phase fraction cells in each group is shown in (**H**). (**D**, **I**) PSCs derived Galectin-1 has an anti-apoptotic effect on PANC-1 *in vitro*. All experiments were repeated three times. ^*^ *p* < 0.05, ^**^ *p* < 0.01, ^#^ *p* > 0.05 vs. wt-Galectin-1 PSCs.

**Figure 4:**
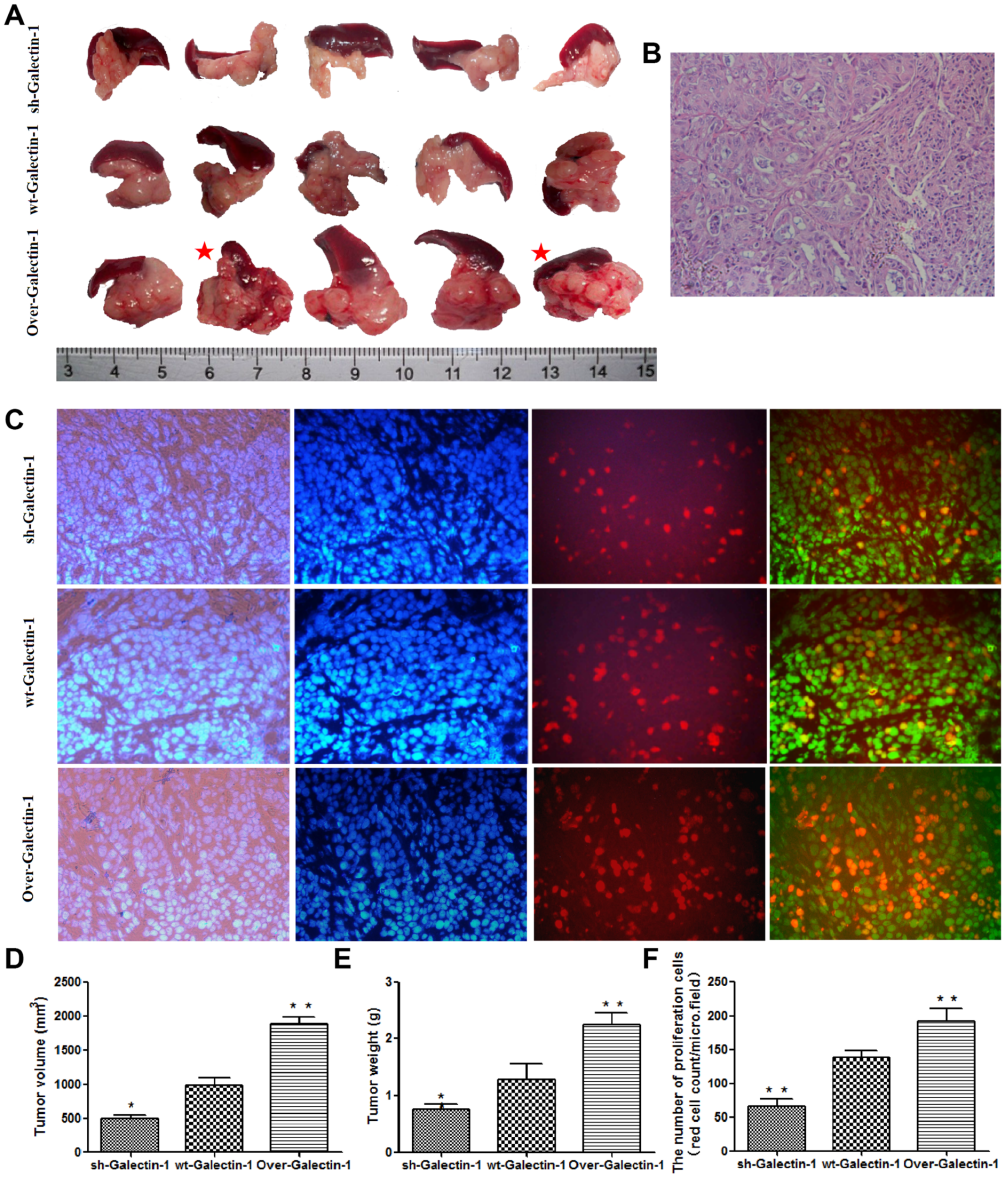
Effect of PSC-derived Galectin-1 on *in vivo* orthotopic xenograft establishment and growth. (**A**) PANC-1 mixed with PSCs were implanted orthotopically into the pancreas of nude mice (*n* = 5). The mice were sacrificed and the xenografts were removed on day 30 after cell implantation. The red star represent cases with liver metastasis. (**B**) H&E staining of samples of orthotopic xenografts in the pancreas of nude mice. (**C**) Proliferating capability of the orthotopic xenograft was evaluated using the EdU incorporation assay, and the number of EdU positive cells per micro field is shown in (**F**). Tumor volume (**D**) and weight (**E**) is expressed as the mean ± SE. ^*^ *p* < 0.05, ^**^ *p* < 0.01, ^#^ *p* > 0.05 vs. wt-Galectin-1 PSCs.

