# Supplementary material for: PSC-derived Galectin-1 inducing epithelial-mesenchymal transition of pancreatic ductal adenocarcinoma cells by activating the NF-κB pathway
Source: Oncotarget. 2017 Sep 23;8(49):86488–502. doi: 10.18632/oncotarget.21212 (PMC5689700; doi:10.18632/oncotarget.21212)
Supplement: Supplementary file 1 [file oncotarget-08-86488-s001.pdf]

PSC-derived Galectin-1 inducing epithelial-mesenchymal transition of pancreatic ductal adenocarcinoma cells by activating the NF-κB pathway

SUPPLEMENTARY MATERIALS

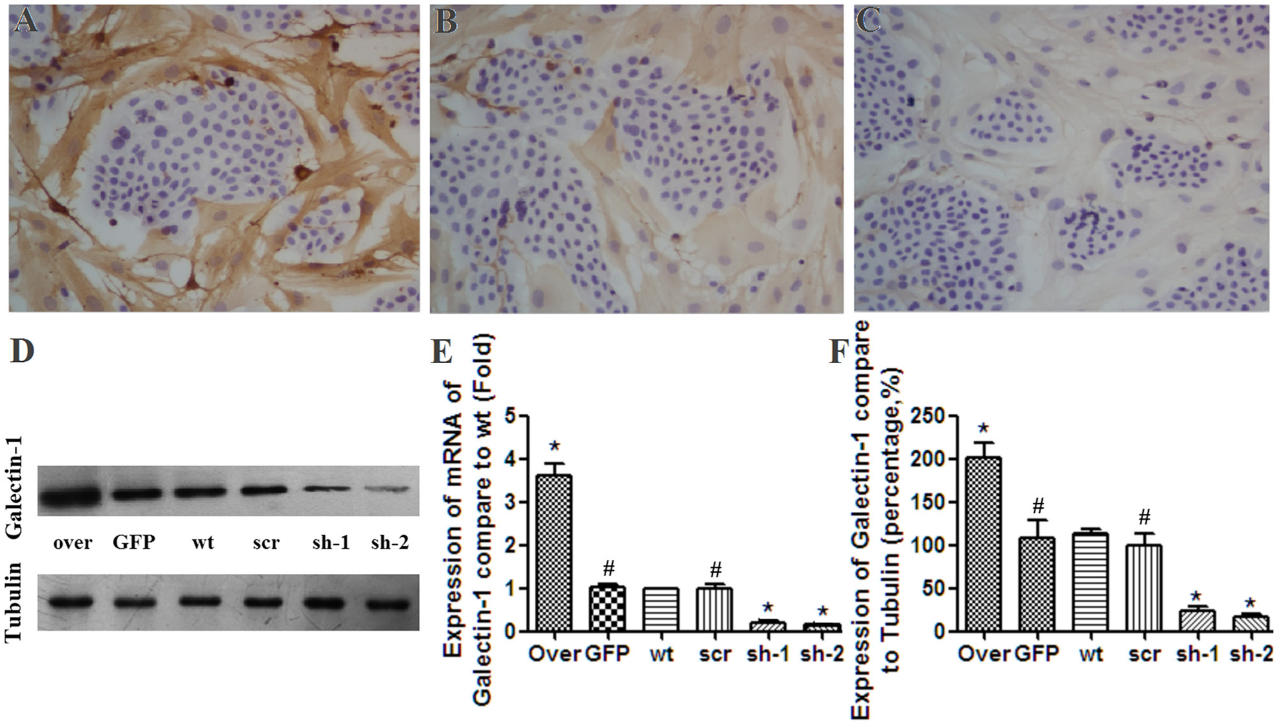

Supplementary Figure 1: Overexpression and knockdown of Galectin-1 in PSCs. Normal PSCs (wt-Galectin-1) and PSCs transduced Galectin-1-overexpressing or knockdown lentivirus were co-cultured with PANC-1. Immunohistochemistry staining was then performed for **(A)** over-Galectin-1 PSCs + PANC-1, **(B)** wt-Galectin-1 PSCs + PANC-1, and **(C)** sh-Galectin-1 PSCs + PANC-1. **(D, F)** Western blot analysis (and quantification) indicating increased Galectin-1 expression in over-Galectin-1 PSCs transduced with the Galectin-1 overexpression lentivirus, and reduced Galectin-1 expression in PSCs transduced with the Galectin-1 knockdown construct #1 or #2 compared to normal and control lentivirus transduced PSCs. **(E)** Quantitative analyses of the different human PSCs. Increased Galectin-1 mRNA expression was observed in the over-Galectin-1 PSCs, and reduced Galectin-1 mRNA expression was observed in the sh-Galectin-1 PSCs. Key: Normal untransduced PSC (wt), and PSCs transduced with GFP control (GFP), Galectin-1 overexpression construct (Over), scrambled shRNA control (scr), shRNA-Galectin-1 #1 (sh-1), and shRNA-Galectin-1 #2 (sh-2). $^{*}p < 0.01$, #$p > 0.05$ (vs. normal PSCs). All experiments were repeated three times.
